# Supplementary material for: Resolving the full spectrum of human genome variation using Linked-Reads
Source: Genome Res. 2019 Apr;29(4):635–45. doi: 10.1101/gr.234443.118 (PMC6442396; doi:10.1101/gr.234443.118)
Supplement: Supplemental Material [file supp_gr.234443.118_Supplemental_longranger-2.2.zip › longranger-2.2.tar.bz2!/lr2.2-public/lib/stan/cmdstan/stan/doc/api/html/dense__e__point_8hpp_source.html]

Stan: src/stan/mcmc/hmc/hamiltonians/dense\_e\_point.hpp Source File


|  |  |
| --- | --- |
|  | Stan  2.5.0  probability, sampling & optimization |


- Main Page
- Related Pages
- Namespaces
- Classes
- Files

- File List
- File Members

All Classes Namespaces Files Functions Variables Typedefs Enumerations Enumerator Friends Macros Pages

- src
- stan
- mcmc
- hmc
- hamiltonians

dense\_e\_point.hpp

Go to the documentation of this file.

1 #ifndef STAN\_\_MCMC\_\_DENSE\_\_E\_\_POINT\_\_BETA

2 #define STAN\_\_MCMC\_\_DENSE\_\_E\_\_POINT\_\_BETA

3

4 #include <stan/mcmc/hmc/hamiltonians/ps\_point.hpp>

5

6 namespace stan {

7

8  namespace mcmc {

9

10  // Point in a phase space with a base

11  // Euclidean manifold with dense metric

12  class dense\_e\_point: public ps\_point {

13

14  public:

15

16  dense\_e\_point(int n): ps\_point(n), mInv(n, n) {

17  mInv.setIdentity();

18  };

19

20  Eigen::MatrixXd mInv;

21

22  dense\_e\_point(const dense\_e\_point& z): ps\_point(z), mInv(z.mInv.rows(), z.mInv.cols()) {

23  fast\_matrix\_copy\_<double>(mInv, z.mInv);

24  }

25

26  void write\_metric(std::ostream\* o) {

27  if(!o) return;

28  \*o << "# Elements of inverse mass matrix:" << std::endl;

29  for(int i = 0; i < mInv.rows(); ++i) {

30  \*o << "# " << mInv(i, 0) << std::flush;

31  for(int j = 1; j < mInv.cols(); ++j)

32  \*o << ", " << mInv(i, j) << std::flush;

33  \*o << std::endl;

34  }

35  };

36

37  };

38

39  } // mcmc

40

41 } // stan

42

43

44 #endif

stan::mcmc::dense\_e\_point::write\_metric

void write\_metric(std::ostream \*o)

**Definition:** dense\_e\_point.hpp:26

stan::mcmc::ps\_point

**Definition:** ps\_point.hpp:16

stan::mcmc::dense\_e\_point::dense\_e\_point

dense\_e\_point(int n)

**Definition:** dense\_e\_point.hpp:16

stan::math::rows

size\_t rows(const Eigen::Matrix< T, R, C > &m)

**Definition:** rows.hpp:12

stan::mcmc::dense\_e\_point::dense\_e\_point

dense\_e\_point(const dense\_e\_point &z)

**Definition:** dense\_e\_point.hpp:22

ps\_point.hpp

stan::mcmc::dense\_e\_point::mInv

Eigen::MatrixXd mInv

**Definition:** dense\_e\_point.hpp:18

stan::math::cols

size\_t cols(const Eigen::Matrix< T, R, C > &m)

**Definition:** cols.hpp:12

stan::mcmc::dense\_e\_point

**Definition:** dense\_e\_point.hpp:12


---

[ Stan Home Page ]

*© 2011–2014,
Stan Development Team.*
